# Superior Vena Cava Syndrome after Radiofrequency Catheter Ablation for Atrial Fibrillation

**DOI:** 10.5935/abc.20170168

**Published:** 2017-12

**Authors:** Maria Luciana Zacarias Hannouche da Trindade, Ana Clara Tude Rodrigues, Cristiano Faria Pisani, Rafael Bonafim Piveta, Samira Saady Morhy, Maurício Ibrahim Scanavacca

**Affiliations:** Hospital Israelita Albert Einstein, São Paulo, SP - Brazil

**Keywords:** Superior Vena Cava Syndrome, Atrial Fibrillation, Catheter Ablation, Arrhythmias, Cardiac

## Introduction

Atrial fibrillation (AF) is the most common sustained cardiac arrhythmia in clinical practice. Its prevalence increases with age, and it is commonly associated with structural heart diseases, leading to hemodynamic and thromboembolic complications with significant economical implications and increasing morbimortality.^1,2^ The AF ablation techniques, when carried out by experienced operators, have proved to be safe and to yield quite satisfactory results, but in some patients it is necessary to approach extra-venous focuses, such as the left atrial appendage, coronary sinus and superior vena cava.^3^ AF ablation is a complex procedure, not without risks. Important complications have been reported, including cerebrovascular accidents, pulmonary vein stenosis and atrioesophageal fistula.^4,5^ We report here a case of superior vena cava syndrome after radiofrequency ablation for AF.

### Case report

Female patient, 60 years old, with mild chronic obstructive pulmonary and pre-syncope episodes, had her first AF episode in July, 2012 and remained asymptomatic until January, 2013, when she had a new AF episode, reversed with amiodarone. Since then, she had recurrent AF episodes, in spite of the use of amiodarone and beta blockers. She was referred for invasive treatment of AF (radiofrequency ablation of the pulmonary veins) and was thus admitted to hospital to undertake the procedure. Transesophageal echocardiography was performed prior to the procedure and showed mild to moderate dilation of the left atrium, thickened mitral valve with prolapse of both cusps and moderate to important regurgitation. There was mild tricuspid regurgitation, with a maximal systolic pulmonary pressure estimated at 46 mmHg; Both atria and respective appendages were free from thrombi, with the left atrial appendage contracting normally (left atrial appendage velocity was estimated at 0.60 m/s); pulmonary venous anatomy and drainage was normal. The electrophysiological procedure was carried out as usual, with the patient under general anesthesia and with the placement of an esophageal thermometer. After the introduction of 3 multi-pole catheters through the femoral vein, they were placed in the coronary sinus and in the left atrium, after a double transeptal puncture. The patient, who was in AF at the start of the procedure, underwent circumferential isolation of the pulmonary veins using an irrigated catheter (Thermocool, Biosense & Webster) under the guidance of an electroanatomic mapping system (CARTO®). During the applications of radiofrequency (RF) to the right pulmonary veins, the AF was reversed to sinus rhythm. After the isolation of the pulmonary veins, it was decided to attempt isolation of the superior vena cava (SVC). The applications on the superior vena cava were carried out using irrigated catheter, with a potency of 30w. These applications were made in a segmented manner, guided by a Lasso catheter. The phrenic nerve was mapped out with high output stimulation and no applications were made to the capture sites. After the procedure, the patient was awaken from the anesthesia and sent to her room.

**Figure 1:**
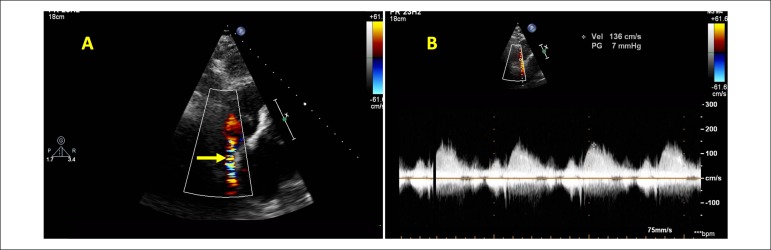
A) Echocardiographic subcostal view with color flow mapping, showing turbulent flow in SVC, one day after the ablation. B) Continuous wave Doppler tracings showing increased velocity in the SVC. SVC: superior vena cava.

**Figure 2:**
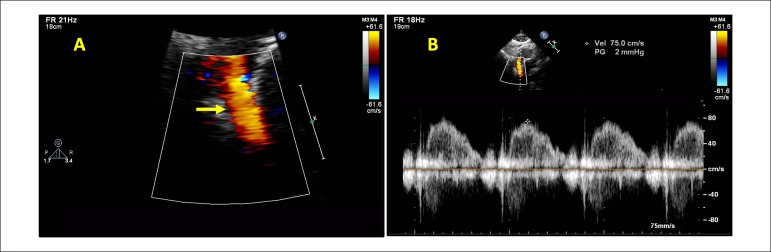
A) Subcostal view with color flow mapping showing laminar flow in the SVC, after the treatment. B) Pulsed-wave Doppler tracings showing normal velocity in the SVC. SVC: superior vena cava.

One day after the procedure, the patient presented with facial edema and flushing of the cheeks and edema of the upper limbs. She also reported pressure on her head and neck, with the symptoms exacerbated when laying horizontally and leaning her head forward, suggesting a superior vena cava syndrome (SVCS). A transthoracic echocardiogram (TTE) was carried out, with similar preprocedural findings, except for the presence of turbulent flow in the SVC at its entry in the right atrium, with Doppler velocity at 136 cm/s (Figure 1), confirming the hypothesis of a SVCS. The patient was treated with hydrocortisone EV, followed by prednisone 60 mg per day, for 13 days, with complete improvement of the symptoms, and was discharged after 7 days. A TTE performed 2 weeks later did not show increased velocity in the SVC (75 cm/s) (Figure 2).

A year later, the patient underwent a new procedure, that showed evidence of electric reconnection of the superior vena cava and the pulmonary veins, thus isolating pulmonary veins was again attempted . An atrial tachycardia originating from the left atrial appendage was mapped out, with no need for applications to the superior vena cava. No difficulty in manipulating catheters at the superior vena cava was observed.

## Discussion

SVCS results from any condition that leads to the obstruction of its blood flow. The obstruction may be caused by the invasion or external compression of the SVC by a pathological process involving the right side of the lung, the lymph nodes and other structures of the mediastinum, or due to thrombosis inside the SVC. In some cases, both external compression and thrombosis may coexist.^6^

More recently, the incidence of SVCS due to thrombosis has increased especially due to the use of intravascular devices, such as central venous catheters and pacemaker wires. Benign causes currently represent 20 to 40% of the SVCS cases.

The rapidity of onset of symptoms and signs from SVC obstruction is dependent upon the rate at which complete obstruction of the SVC occurs in relation to the recruitment of venous collaterals.

Interstitial edema of the head and neck is visually impressive, but usually of very little clinical importance. However, the edema may obstruct the lumen of the nasal cavity and of the larynx, and potentially compromise the function of the larynx and pharynx, causing dyspnea, wheezing, cough, hoarseness and dysphagia.

Vascular complications are among the most common adverse events related to AF ablation, most likely due to the need for anticoagulation during and immediately after the procedure. These complications include hematoma at the site of catheter insertion, pseudo-aneurysm, arteriovenous fistula, or retroperitoneal bleeding.

Transitory occlusion of the SVC is rare, and has only been reported after catheter ablation of inappropriate sinus tachycardia,^7^ with no reports of this complication resulting from catheter ablation of AF ablation yet. It is assumed that the mechanisms associated with the RF-induced venous occlusion leads to intimal proliferation, substitution of the necrotic muscle by collagen, endovascular contraction and rupture and thickening of the internal elastic lamina.^8^

Venous structures of smaller diameter, such as the coronary sinus and the pulmonary veins, may be subject to an even greater risk of occlusion as a result of ablation procedures carried out in the adjacent tissue.^9^

In this patient, the RF application inside the superior vena cava, performed to eliminate this possible trigger, probably caused the formation of a significant edema in the junction of the SVC with the right atrium. This edema is more marked in the area that receives the RF, but it also occurs throughout its entire circumference, presumably by the propagation of the interstitial edema through the contiguous tissue. This edema of the tissues, resulting in the narrowing of the SVC-right atrium junction, may persist throughout the entire period of time of the ablation procedure, but seems to be resolved within weeks or months. Studies in animals have shown that the thickening of the atrial tissue after RF application increases with time, and persists for at least 150 min.^8^ Even though the complete occlusion of the SVC was not observed in this patient, the characteristics of the Doppler flow observed in the junction between the SVC and the right atrium lead to the conclusion that the edematous tissue induced by the RF is a plausible mechanism for such complication.
